# Prolactin may serve as a regulator to promote vocal fold wound healing

**DOI:** 10.1042/BSR20200467

**Published:** 2020-07-22

**Authors:** Haizhou Wang, Xueyan Li, Jieyu Lu, Paul Jones, Wen Xu

**Affiliations:** 1 Department of Otolaryngology-Head and Neck Surgery, Beijing Tongren Hospital, Capital Medical University, China; 2 Key Laboratory of Otolaryngology-Head and Neck Surgery, Ministry of Education of China, Beijing, China; 3 Department of Biology Solution, AIC LLC, Rockville, MD 20850, U.S.A.

**Keywords:** cell function, gene expression, signaling pathway, Vocal fold injury

## Abstract

Reduced prolactin (PRL) has been shown to delay wound healing with a limited understanding of the underlying mechanisms. Here, we aim to explore the role of PRL in the repair of vocal fold (VF) injury. A microarray was used to detect the expressed levels of PRL in rat VF tissue at 1, 4, and 8 weeks after VF injury compared with normal uninjured rats. Then, a systematic bioinformatics analysis has been conducted to explore the literature-based biology network and signaling pathways involved in the repair of VF injury. The expression of PRL was significantly decreased in all VF injury groups (week 1, 4, and 8) compared with the control group (*F* stats = 280.34; *P*=4.88e-14), with no significant difference among the three VF injury groups (*F* stats = 1.97; *P*=0.18). Wounding has been shown to interfere with both PRL-promoting and inhibiting pathways that were involved in wound healing, including 11 PRL inhibitors and 6 PRL promoters. Our results reveal decreased PRL expression levels in VF injury, which is not in favor of the wound healing. The pathways identified may help in understanding the role of PRL as a treatment target for VF wound healing.

## Introduction

The vocal fold, as a vocal organ, produces sounds by its viscoelasticity, which is largely dependent on the structure of the extracellular matrix (ECM) [1]. Excessive injury or stimuli to VFs leads to disordered deposition of ECM in the lamina propria and abnormal massive hyperplasia of the fibrous tissue. Fibrous scars result from the disordered arrangement of fibers, causing irreversible changes, and persistent pararthria [2–4]. Therefore, novel treatment strategies and therapeutic approaches need to be urgently developed.

VF healing is a dynamic process that can last for several months [5,6]. Many genes and proteins have been suggested to play various roles at different regeneration stages after injury. Previous studies have shown that inflammatory responses occur at an early stage after VF injury, where there is a massive proliferation of fibroblasts with intensive secretion and deposition of ECM [5]. These activities reach a peak at 3 to 7 days after injury, and their intensity begins to decline 15 days later [4]. A study based on the dermal system has shown that ECM remodeling during the repair of injury can last for several months [6]. However, so far, the genetic changes and related signaling pathways during the healing process after VF injury remains largely unclear.

Recently, prolactin (PRL) has been suggested to play an important role in the cutaneous wound healing to control the excessive reepithelialization [7], which suggested a possible role of PRL receptor antagonists in the promotion of epidermal repair after human wounding. Prolactin has also been suggested to promote wound healing through the regulation of multiple cell types’ proliferation [8]. In the present study, we first tested the expression change of PRL in the case of VF wound at different time points after injury, using a rat model. Then, we conducted a systematic signaling pathway analysis to explore the underlying mechanisms regarding the regulation of intracutaneous PRL expression by wounding. Our results may add new insights into the understanding of the VF healing process that is needed for the treatment of VF wound.

## Materials and methods

### Animal experiments

Male Sprague–Dawley (SD) rats aged 14–16 weeks and weighing 400–450 g underwent unilateral or bilateral VF injury using procedures described in an earlier study [9]. The VF was injured by separating and removing the lamina propria from the thyroarytenoid muscle. Twenty animals were randomly divided into four experimental groups (five animals in each group) based on time of sacrifice: uninjured control, 1, 4, and 8 weeks after injury. During the study, rats in all the experimental groups were anesthetized by intraperitoneal injection of pentobarbital sodium (Sinopharm Chemical Reagent Co., Ltd, Shanghai, China) according to the rat weight at 50 mg/kg and finally killed by intraperitoneal injection of the overdose of 10% chloral hydrate (Sinopharm Chemical Reagent Co., Ltd, Shanghai, China). The animal experiments in the present study were conducted at Peking University and approved by Peking University Animal Care and Use Committee (LSC-ZhangY-1; license permit: 1116012800123).

### Expression profile of PRL in different groups

Total RNA was extracted from VFs using TRIzol Reagent (Life Technologies, Carlsbad, CA, U.S.A.) and purified with an RNeasy mini kit (Qiagen, Valencia, CA, U.S.A.). Biotinylated cDNA was prepared according to the standard Affymetrix protocol from 250 ng of total RNA using an Ambion® WT Expression Kit. Following labeling, fragmented cDNA was hybridized for 16 h at 45°C with the Clariom™ S Assay (rat, Affymetrix). GeneChips were washed and stained in the Affymetrix Fluidics Station 450. All arrays were scanned using an Affymetrix® GeneChip Command Console (AGCC), which was installed in a GeneChip® Scanner 3000 7G.

The row data (.cel) were normalized using TAC software (Transcriptome Analysis Console, Version 4.0.1) with the Robust Multichip Analysis (RMA) algorithm using Affymetrix default analysis settings and global scaling as normalization method. The values presented are log_2_ RMA signal intensity. The microarray data discussed in this article are publicly available at NCBI Gene Expression Omnibus (GEO) under accession number GSE139383.

### Experiment data analysis

One-way analysis of variance (ANOVA) was used to compare the expression levels of PRL in four groups: (1) one week after VF injury versus uninjured control; (2) four weeks after VF injury versus uninjured control; (3) 8 weeks after VF injury versus uninjured control. *F* value and *P*-value were reported. The analysis was conducted using Matlab (version R2017a).

### Bioinformatics analysis

To explore the possible mechanisms regarding how wounding regulates PRL expression, we conducted a large scale literature data mining by using Pathway Studio (www.pathwaystudio.com). The upstream genetic inhibitors and promoters of PRL were firstly identified; then, we collected the downstream genetic targets of wounding. Based on these results, we built the functional networks connecting wound, PRL and wound healing. Specifically, we built the pathways where the wound promotes PRL inhibitors and inhibits PRL promoters. We also identified the relationship between PRL and wound healing. Each of the relationships identified within the pathways was supported by one or more references, where were provided in the Supplementary Materials. These references were processed by using Natural Language Processing (NLP) technique that covers more than 24 million PubMed abstracts and over 3.5 million Elsevier and third party full-test papers (Database Release Notes of Pathway Studio Version 12.3.0.16).

To understand the profile of the genes identified in the above functional networks, we conducted a gene set enrichment analysis (GSEA). The GSEA was conducted against the Pathway Studio pathways and gene ontology (GO) terms. Significantly enriched pathways and corresponding statistics were reported.

## Results

### PRL expression

**Figure 1:**
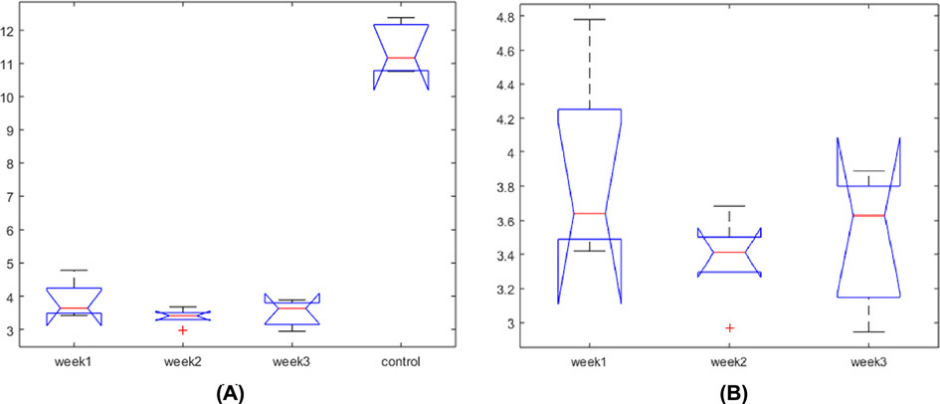
ANOVA results comparing expression levels of PRL in four different groups: (1) one week after vocal fold injury; (2) four weeks after vocal fold injury; (3) eight weeks after vocal fold injury, and (4) uninjured control (**A**) ANOVA results of comparing four groups; (**B**) ANOVA results of comparing three groups.

As shown in Figure 1, the expression levels of PRL were significantly lower in all injury groups (week 1, 4, and 8) compared with the healthy control group (*F* stats = 280.34; *P*=4.88e-14). On the other hand, the three injury groups demonstrated no significant difference among each other (*F* stats = 1.97; *P*=0.18). In addition, the PRL in the 2-week rat group was lower than that of the first-week group (3.38 ± 0.26 vs. 3.88 ± 0.56), which was consistent with previous reports that serum PRL levels decreased according to the survival period of blunt injury [10]. However, PRL expression level demonstrated a slight recovery in the 8-week group (3.45 ± 0.40), which may reflect the progress of the wound healing.

### Wounding promotes PRL inhibitor

**Figure 2:**
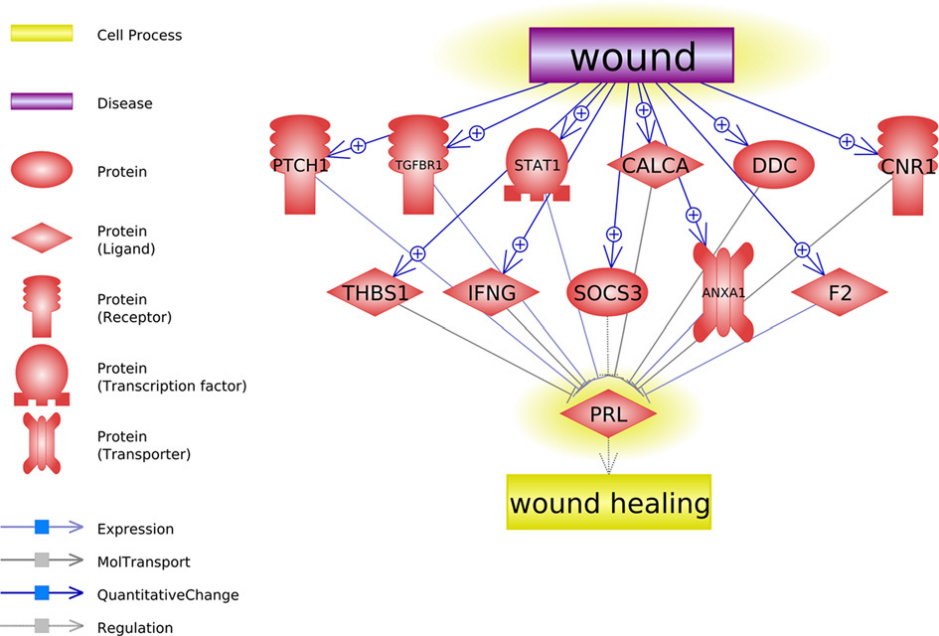
Network showing wounding promotes PRL inhibitors The network was built using Pathway Studio. Each relation was supported by one or more scientific references.

As shown in Figure 2, we identified 11 PRL inhibitors that got promoted by wounding. On the other hand, PRL participates in wound healing and the regulation of cell proliferation for a variety of cell types. Each of these relations identified was supported by at least one previous scientific study. The detailed information of supporting references of the relations presented in Figure 2, including the relation type, reference title, and the sentences where a relationship has been identified, have been presented in Supplementary Materials: Ref_PRL_inhibitors.

Using the 11 genes connection wounding and PRL (Figure 2) as input, 15 pathways got significantly enriched and listed in Table 1. Most of these pathways were involved in interferon-gamma signaling, epithelial cell and endothelial cell proliferation, wound healing, and growth factor response. Our results showed that wounding might inhibit the secretion of prolactin through the positive regulation of these pathways.

**Table 1:** GSEA results for the PRL–inhibitor network

Name	GO ID	# of Entities	Overlap	*P*-value	Jaccard similarity
GO: regulation of interferon-γ-mediated signaling pathway	0060334	28	3	0.00045	0.083
GO: regulation of response to interferon-γ	0060330	28	3	0.00045	0.083
GO: cellular response to growth factor stimulus	0071363	397	5	0.00075	0.012
GO: response to growth factor	0070848	448	5	0.0011	0.011
GO: regulation of epithelial cell proliferation	0050678	454	5	0.0012	0.011
GO: regulation of epithelial cell differentiation	0030856	192	4	0.0013	0.02
GO: aging	0016280	493	5	0.0015	0.010
GO: negative regulation of epithelial cell proliferation	0050680	200	4	0.0015	0.019
GO: negative regulation of epithelial cell differentiation	0030857	62	3	0.0021	0.043
GO: positive regulation of wound healing	0090303	68	3	0.0027	0.039
SOCS in negative feedback regulation in myocarditis	NONE	21	3	0.0035	0.10
GO: cell proliferation involved in metanephros development	0072203	8	2	0.0035	0.12
GO: positive regulation of response to wounding	1903036	85	3	0.003	0.032
GO: negative regulation of cytokine production	0001818	301	4	0.0041	0.013
GO: negative regulation of endothelial cell proliferation	0001937	87	3	0.0041	0.032

### Wounding suppresses PRL–promoters

**Figure 3:**
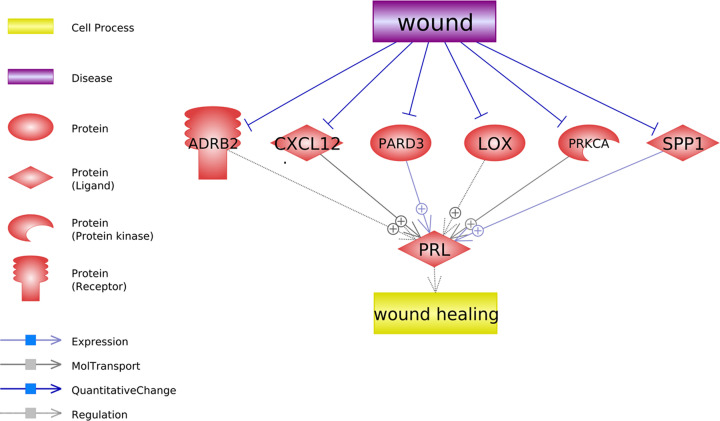
Network showing wounding suppresses PRL promoters The network was built using Pathway Studio. Each relation was supported by one or more scientific references.

In addition to the PRL inhibiting pathway, we also identified six PRL promoters that got inhibited by wounding, as shown in Figure 3. Each of these relations identified was supported by at least one previous scientific study. For details of the references supporting the relations, including the relation type, reference title, and the sentences where a relationship has been identified, please refer to Supplementary Materials: Ref_PRL_promoters.

Using the six genes connection wounding and PRL (Figure 3) as input, ten pathways got significantly enriched and listed in Table 2. Most of these pathways were involved in developmental growth regulation, endothelial cell proliferation, response to wounding, and steroid hormone. Our results showed that wounding might suppress the secretion of prolactin through the negative regulation of these pathways.

**Table 2:** GSEA results for the PRL–promoter network

Name	GO ID	# of Entities	Overlap	*P*-value	Jaccard similarity
GO: regulation of developmental growth	0048638	445	4	0.023	0.0089
GO: response to steroid hormone	0048545	418	4	0.023	0.0095
GO: regulation of endothelial cell proliferation	0001936	204	3	0.046	0.014
GO: regulation of response to wounding	1903034	199	3	0.046	0.015
GO: apical part of cell	0045177	132	3	0.026	0.022
GO: positive regulation of calcium ion import	0090280	26	2	0.046	0.065
GO: positive regulation of bone remodeling	0046852	26	2	0.046	0.065
GO: positive regulation of bone resorption	0045780	26	2	0.046	0.065
GO: regulation of platelet aggregation	0090330	20	2	0.038	0.080
GO: induction of positive chemotaxis	0050930	20	2	0.038	0.080

## Discussion

Healing of VF injury is a dynamic process, and it is divided into several stages in which different cell functions and signaling pathways are involved, with limited understanding of the dynamic changes underlying the healing process. Several studies indicated that prolactin (PRL) might regulate the proliferation of multiple cells and facilitate wound healing [7,8]. Here, we first tested the expression changes of PRL in the case of VF injury using an animal model. Then, we explored large-scale literature-based pathway analysis and gene set enrichment analyses (GSEA) to explore the biology mechanism explaining the activity of PRL during wounding and the healing of wound. Our results may add new insights into our knowledge about the healing process of VF injury and relevant working mechanisms, and provides a reference for new assumptions and targets for treatment.

Our animal model showed that, compared to un-hurt rats, PRL demonstrated significantly low expression levels in all three groups (1-, 4-, and 8-week rats) (*P*<4.89e-14), while there was no significant difference among the three VF injury groups (*P*>0.18). These results were consistent with previous studies that serum PRL levels decreased in blunt injury cases [10]. The longer the blunt injury, the lower the decreased levels of serum PRL. However, with the recovery of the injury, the expression or PRL levels may restore (Figure 1B).

Literature-based pathway analysis showed that, in the case of wounding, multiple PLR inhibitors get activated and promoters depressed, as shown in Figures 2 and 3. Specifically, there were 11 proteins that were simulated by wounding, which could inhibit the section of prolactin. For instance, wounding induces expression of IFNG mRNA and protein by infiltrating T cells and macrophages [11], while increased IFNG and TNFA could inhibit the secretion of prolactin by the anterior pituitary [12]. Moreover, it has been shown that epithelial wounding could induce the expression of cytokine-induced SH2 protein 3 (SOCS3) [13], which inhibits growth hormone and PRL signaling [14]. More pathways and the corresponding references are presented in Figure 2 and Supplementary Materials: Ref_PRL_inhibitors. These pathways may partially explain the mechanisms of the decreased PRL levels in VF injury.

On the other hand, we also identified pathways where six PRL promoters get inhibited by wounding (see Figure 3). It has been shown that the activation of alpha 2 and beta 2 adrenoceptors (ADRB2) could result in the stimulation of prolactin activity [15]. However, the expression of the beta 2 adrenoceptors was shown to decrease right after wounding [16]. Wounding also decreases the expression of CXCL12 in a mice model [17], and CXCL12 has been showed to be a stimulator of PRL-3 expression [18]. More pathways and related references can be found in Supplementary Materials: Ref_PRL_promoters, which could add more insights into the understanding of the possible mechanism of the decreased PRL expression in VF injury.

GSEA results showed that the inhibitors of PRL influenced by wounding were mainly involved in interferon-gamma signaling, epithelial cell and endothelial cell proliferation, wound healing and growth factor responds, which were suggested to associate with VF wound healing [19–22]. These results indicated that VF wound might up-regulate these pathways that influence the secretion of prolactin. Interestingly, the PRL promoters inhibited by wounding were also involved in developmental growth, endothelial cell proliferation, response to wounding. Furthermore, four out of the six PRL-promoters (Figure 3) were found enriched in steroid hormone responding pathways, including SPP1, PRKCA, ADRB2, and LOX (see Table 2 and Supplementary Materials: Pathway_PRL_promoters). It has been suggested that injectable estradiol and dexamethasone might have similar effects on wound healing in VF injuries [23]. These results could help in understanding the association between VF injury and PRL and the possible roles that PRL plays in the VF wound healing.

Although expression data and pathway analysis identified decreased PRL expression in VF injury which is not in favor of wound healing, the finding should be directly tested by comparing the VF injury healing process with/without PRL being activated. This could be a valuable future work.

## Conclusion

Our results showed that the expression of PRL was significantly decreased in VF wounding, which may delay the healing of the wound. The signaling pathways identified may help in the understanding of the relationship between PRL and VF wounding. The results of this study suggested that PRL may be a treatment target for VF wound healing.

## Supplementary Material

Supplementary Materials

## Abbreviations

ADRB2: alpha 2 and beta 2 adrenoceptors
ECM: extracellular matrix
NLP: Natural Language Processing
PRL: prolactin
VF: vocal fold
